# Risk of Aortic Aneurysm and Dissection in Patients with Tuberculosis: A Nationwide Population-Based Cohort Study

**DOI:** 10.3390/ijerph182111075

**Published:** 2021-10-21

**Authors:** Ming-Tsung Chen, Chi-Hsiang Chung, Hung-Yen Ke, Chung-Kan Peng, Wu-Chien Chien, Chih-Hao Shen

**Affiliations:** 1 Division of Pulmonary and Critical Care Medicine, Department of Internal Medicine, Tri-Service General Hospital, National Defense Medical Center, Taipei 11490, Taiwan; a01016233@yahoo.com.tw (M.-T.C.); kanpeng1025@yahoo.com.tw (C.-K.P.); 2 Department of Medical Research, Tri-Service General Hospital, National Defense Medical Center, Taipei 11490, Taiwan; g694810042@gmail.com; 3 School of Public Health, National Defense Medical Center, Taipei 11490, Taiwan; 4 Division of Cardiovascular Surgery, Department of Surgery, Tri-Service General Hospital, National Defense Medical Center, Taipei 11490, Taiwan; drkehy@yahoo.com.tw

**Keywords:** tuberculosis, aortic aneurysm, aortic dissection

## Abstract

Tuberculosis (TB) can cause chronic inflammation. The occurrence of aortic aneurysm (AA) and aortic dissection (AD) may be associated with chronic inflammatory disease, but whether TB increases the risk of AA and AD remains to be determined. This study aimed to investigate the association between TB and the development of AA and AD. We conducted a population-based cohort study using data obtained from the Taiwan National Health Insurance Database. We selected 31,220 individuals with TB and 62,440 individuals without TB by matching the cohorts according to age, sex, and index year at a ratio of 1:2. Cox regression analysis revealed that the TB cohort had a 1.711-fold higher risk of AA and AD than the non-TB cohort after adjustment for sex, age, socioeconomic status, and comorbidities (adjusted hazard ratio = 1.711; 95% confidence interval = 1.098–2.666). Patients with pulmonary, extrapulmonary, and miliary TB had a 1.561-, 1.892-, and 8.334-fold higher risk of AA and AD, respectively. Furthermore, patients with TB at <6 months, 6–12 months, and 1–5 years of follow-up had a 6.896-, 2.671-, and 2.371-fold risk of AA and AD, respectively. Physicians should consider the subsequent development of AA and AD while treating patients with TB.

## 1. Introduction

Tuberculosis (TB), an infectious disease caused by *Mycobacterium tuberculosis*, is one of the major causes of death worldwide. In 2019, approximately 10 million individuals were diagnosed with TB, and 1.4 million individuals died because of TB [1]. In Taiwan, the incidence of TB was 37 cases per 100,000 people and that of TB-related deaths was 2.3 per 100,000 people in 2019 [2]. TB typically affects the lungs and results in cavitation, fibrosis, bronchiectasis, and impaired pulmonary function [3]. TB that affects other organs, such as the lymph nodes, pleura, gastrointestinal tract, bones, urogenital tract, and central nervous system, is known as extrapulmonary TB and accounts for 20–25% of all TB cases [4].

Aortic aneurysm (AA) is the permanent dilation of the aorta and most commonly occurs in the infrarenal and proximal thoracic aortic regions. Most AAs are asymptomatic, but progressive enlargement of the aneurysm increases the risk of dissection and rupture [5]. Aortic dissection (AD) is defined as a tear in the inner layer of the aortic wall that leads to the formation of true and false lumens. AD is a relatively uncommon, life-threatening vascular disease with an annual incidence ranging from 3 to 6 cases per 100,000 persons [6,7]. AA and AD have common risk factors, including smoking, hypertension (HTN), genetic connective tissue disorders, male sex, older age, and vascular inflammation caused by infection or atherosclerosis [6,8,9,10]. Infection can lead to arterial wall aneurysmal degeneration, referred to as mycotic aneurysm, a term first coined by William Osler in 1885 [11], and constitutes 0.6–2% of all arterial aneurysms. A review of the literature on mycotic AA from 2000 to 2018 found that the most common microorganism isolated in the disease was *Salmonella* spp. (33.4%), followed by *Staphylococcus* spp. (15.6%), *Streptococcus* spp. (10.4%), and *Escherichia coli* (3.1%). However, *Mycobacteria* spp. only constituted 2% of all cases [12].

Previous studies demonstrated that patients with TB have a higher risk of developing systemic diseases such as liver cirrhosis, Parkinson’s disease, sarcoidosis, systemic lupus erythematosus, acute coronary syndrome, and peripheral arterial disease [13,14,15,16,17,18]. Inflammation caused by immune response activation and cytokine induction is a crucial pathogenic mechanism of TB infection [19]. Chronic inflammation also plays an essential role in atherosclerosis, which leads to the development of various cardiovascular diseases [20,21]. Because chronic inflammation increases the risk of developing AA and AD [22,23], and as TB may cause chronic inflammation, we hypothesized that patients with TB have an increased risk of developing AA and AD. We conducted this nationwide population-based cohort study to assess the association between these two diseases.

## 2. Materials and Methods

### 2.1. Data Source

The data assessed in this study were obtained from the Longitudinal Health Insurance Database (LHID), a subset of the Taiwan National Health Insurance (NHI) Research Database (NHIRD). NHIRD maintains data on all claims of the beneficiaries of the Taiwan NHI program and uses the International Classification of Diseases, Ninth Revision, Clinical Modification (ICD-9-CM) system for recording diagnoses. The Taiwanese government established the NHI in 1995 as a single-payer compulsory program for all 23 million Taiwanese people. For the protection of privacy, NHIRD removes identifying information and assigns an anonymous number before releasing patient records for research purposes. This study was approved by the Institutional Review Board of the Tri-Service General Hospital, National Defense Medical Center (TSGHIRB No. B-110-21).

### 2.2. Sampled Patients

**Figure 1:**
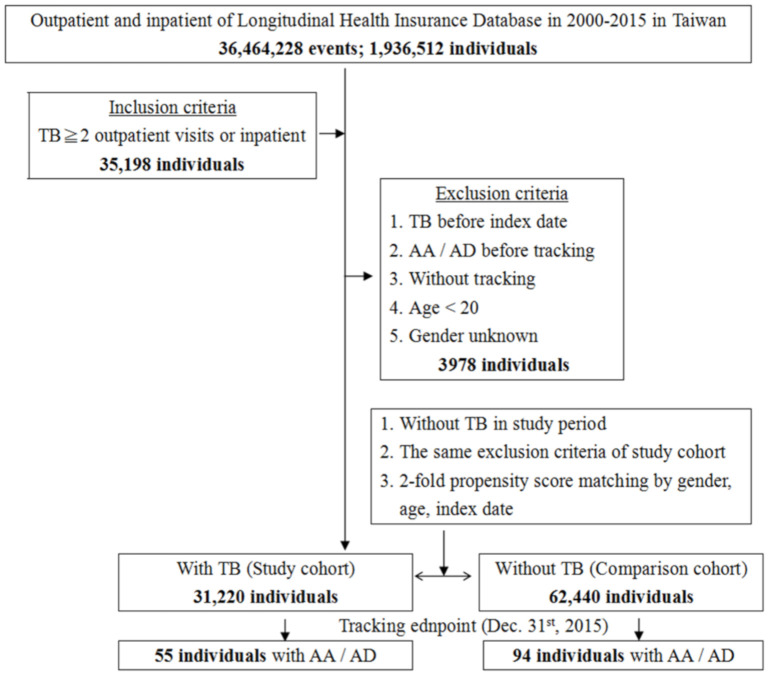
Patient selection flowchart. TB = tuberculosis, AA = aortic aneurysm, AD = aortic dissection.

We conducted a nationwide population-based cohort study to investigate the incidence of AA and AD among patients with TB and identify the associated risk factors. Records of patients aged ≥ 20 years who were diagnosed with TB (ICD-9-CM 010–018) between 2000 and 2015 were obtained from the LHID (Figure 1). The date of diagnosis served as the index date. The follow-up period was defined as the interval from the index date to the date of AA and AD diagnosis. We excluded patients with a history of TB, AA, or AD (ICD-9-CM 441.0–441.9) before the index date, those aged < 20 years, and those with incomplete medical information. We randomly selected the TB and non-TB cohorts with the same exclusion criteria from the LHID and matched them by frequency according to their age, sex, and index year at a ratio of 1:2. The TB cohort was subdivided into pulmonary, extrapulmonary, and miliary groups for subgroup analyses. Furthermore, the TB cohort was classified according to different sites of AA and AD, namely, thoracic (ICD-9-CM 441.01, 441.1, 441.2), abdominal (ICD-9-CM 441.02, 441.3, 441.4), thoracoabdominal (ICD-9-CM 441.03, 441.6, 441.7), and unspecified sites (ICD-9-CM 441.00, 441.5, 441.9).

### 2.3. Outcome Measurement and Comorbidities

All the patients were followed up from the index date until the first diagnosis of AA/AD, death, withdrawal from the NHI program, or 31 December 2015. The study included baseline comorbidities such as diabetes mellitus (DM; ICD-9-CM 250), HTN (ICD-9-CM 401–405), hyperlipidemia (ICD-9-CM 272), ischemic heart disease (IHD; ICD-9-CM 410–414), chronic obstructive pulmonary disease (COPD; ICD-9-CM 490–496), stroke (ICD-9-CM 438), chronic kidney disease (CKD; ICD-9-CM 585), peripheral arterial occlusion disease (PAOD; ICD-9-CM 443.9), and obesity (ICD-9-CM 278.0–278.1).

### 2.4. Statistical Analysis

We compared the distribution of categorical characteristics and baseline comorbidities between patients with and without TB using the chi-square test. In addition, we compared continuous variables between the cohorts using Student’s *t*-test. We used the Kaplan–Meier method to estimate the cumulative incidences of AA and AD and performed the log-rank test to examine the differences between the cohorts. The incidence rate ratios (IRR) of AA and AD in both cohorts were estimated using Poisson regression analysis. Cox regression analysis was used to estimate the adjusted hazard ratios (HR) for the development of AA and AD after adjusting for sex, age, insured premium, urbanization level, and comorbidities, such as DM, HTN, hyperlipidemia, IHD, COPD, stroke, CKD, PAOD, and obesity. All the analyses were conducted using SPSS software, version 26 (SPSS Inc., Chicago, IL, USA). *p*-values < 0.05 indicated statistical significance for a two-sided test.

## 3. Results

We included 31,220 patients in the TB cohort and 62,440 patients in the non-TB cohort. The distributions of age and sex were similar between the cohorts. The mean follow-up periods for the TB and non-TB cohorts were 9.52 ± 11.02 and 10.04 ± 9.18 years, respectively. The patients were predominantly male (71.17%) and aged ≥ 70 years (46.41%). The TB cohort had a higher prevalence of DM and COPD; lower prevalence of HTN, hyperlipidemia, IHD, stroke, CKD, and obesity; and a lower urbanization level than the non-TB cohort (Table 1).

**Figure 2:**
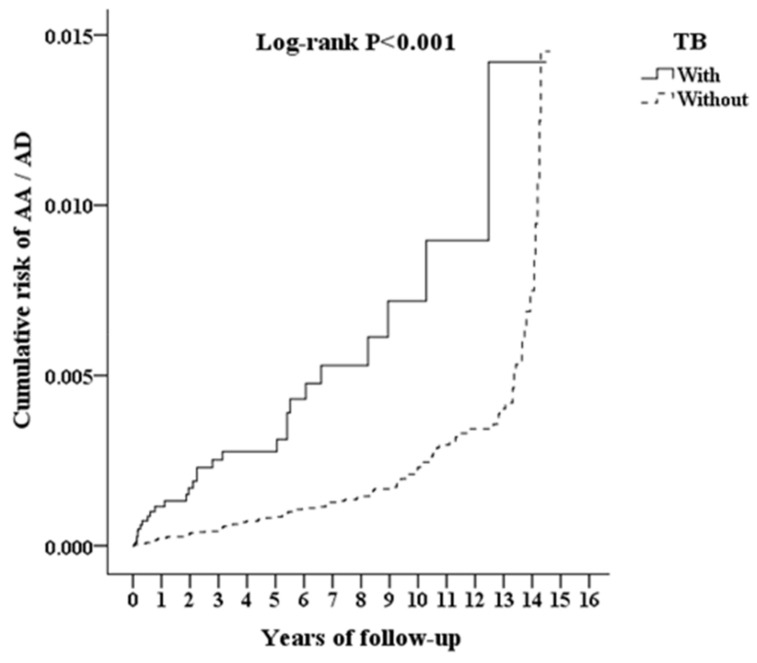
Kaplan–Meier curve for the cumulative risk of aortic aneurysm and aortic dissection due to tuberculosis. TB = tuberculosis, AA = aortic aneurysm, AD = aortic dissection.

Patients with TB had a higher risk for further development of AA and AD than patients without TB (adjusted HR = 1.711; 95% CI = 1.098–2.666; *p* < 0.001) in Table 2. The risk of AA and AD was higher in patients with HTN, IHD, and stroke than in patients without these comorbidities. As shown in Figure 2, the cumulative incidence of AA and AD in subsequent years was higher in the TB cohort than in the non-TB cohort (log-rank test, *p* < 0.001).

Table 3 shows the stratified analysis performed based on demographic factors and comorbidities. The incidence of AA and AD was higher in the TB cohort than in the non-TB cohort (0.185 vs. 0.15 per 1000 person-years), and the overall IRR of AA and AD was 1.235-fold higher in the TB cohort than in the non-TB cohort. The sex-specific relative risk of AA and AD was higher in patients with TB than in those without TB regardless of sex (males, adjusted HR = 1.738; 95% CI = 1.115–2.702; females, adjusted HR = 1.616; 95% CI = 1.034–2.518). The age-specific relative risk of AA and AD was higher in patients with TB regardless of age group. The age-specific incidence of AA and AD increased in both cohorts, with the highest rate in patients with TB aged ≥ 70 years (0.244 per 1000 person-years). In the comorbidity-specific analysis, the adjusted HR for AA and AD was higher in patients with TB than in those without TB, regardless of the presence of DM, IHD, COPD, stroke, or CKD.

We categorized the TB cohort into pulmonary, extrapulmonary, and miliary TB subgroups based on ICD-9-CM codes. Table 4 shows the incidence and adjusted HR of AA and AD for the different types of TB. All TB subgroups had a higher risk of developing AA and AD than patients without TB (pulmonary: adjusted HR = 1.561, 95% CI = 1.005–2.431; extrapulmonary: adjusted HR = 1.892, 95% CI = 1.214–2.936; and miliary: adjusted HR = 8.334, 95% CI = 5.348–12.896).

We conducted a subgroup analysis of the incidence and adjusted HR of the different sites of AA and AD. The adjusted HR was higher in the TB cohort than in the non-TB cohort for AA and AD occurring in the thoracic (adjusted HR = 1.615; 95% CI = 1.044–2.511), abdominal (adjusted HR = 1.588; 95% CI = 1.025–2.469), thoracoabdominal (adjusted HR = 2.910; 95% CI = 1.876–4.557), and unspecified sites (adjusted HR = 1.823; 95% CI = 1.175–2.843) (Table 5).

Table 6 presents the incidence and adjusted HR of AA and AD in both cohorts stratified by the follow-up period. The adjusted HR was higher in the TB cohort than in the non-TB cohort for all follow-up period brackets (within 6 months: adjusted HR = 6.896, 95% CI = 5.010–8.226; 6–12 months: adjusted HR = 2.671, 95% CI = 1.675–3.145; and 1–5 years: adjusted HR = 2.371, 95% CI = 1.486–2.884).

## 4. Discussion

This is the first nationwide, population-based cohort study to investigate the risk of AA and AD in patients with TB by subgroup analyses. The overall finding was that patients with TB, overwhelmingly, have an increased risk and incidence of AA and AD compared with patients without TB, regardless of sex, age, socioeconomic status, and comorbidities. These findings strengthen the possibility of TB being an independent factor for AA and AD. Widespread hematogenous dissemination of *M. tuberculosis* (miliary TB) can affect multiple organs, such as the lungs, liver, spleen, and central nervous system. Life-threatening cardiovascular complications have been identified in patients with miliary TB [24]. A previous study showed that disseminated TB had a higher serum procalcitonin level than non-disseminated TB (0.75 ± 0.79 versus 0.14 ± 0.39 ng/mL; *p* < 0.0001), which was correlated with the severity of inflammation and poor prognosis [25]. In our study, compared with patients without TB, patients in the subgroup with miliary TB had the highest adjusted HR of AA and AD (adjusted HR, 8.334; 95% CI, 5.348–12.896), which highlighted the role of systemic inflammation in the development of AA and AD.

Tuberculous mycotic AAs can develop through direct invasion of the aortic intima, seeding adventitia from the vasa vasorum, and most commonly, direct extension from an adjacent focus of TB infection [26]. In addition to direct aortic damage by *M. tuberculosis*, we propose two possible mechanisms of how TB causes AA and AD. First, TB infection could increase the expression of matrix metalloproteinases (MMPs) via multiple intracellular signaling cascades, mainly NF-κB, p38, and the mitogen-activated protein kinase pathway, and reflect the disease severity [27,28]. MMPs are zinc-dependent endopeptidase proteins that can degrade and fragment various components of the extracellular matrix (ECM), such as collagen, elastin, fibronectin, and laminin. AA and AD occur as a result of disruption of the aortic wall integrity caused by ECM degradation. MMP-1, MMP-2, and MMP-9 have been shown to be fundamental in the development of AA and AD [29,30]. Previous studies showed that high plasma MMP-9 levels are associated with increased AA and AD formation and especially aneurysm rupture [22,31,32]. Hence, elevated MMP levels may contribute to TB-induced AA and AD.

Second, atherosclerosis may cause mechanical weakening of the aortic wall, compensatory lumen enlargement, and activation of inflammation-inducing proteolytic enzymes, resulting in AA formation [33,34]. Furthermore, an ulcerating atherosclerotic plaque that penetrates through the elastic lamina into the media can cause intramural hematoma, dissection, or rupture [35,36]. Various microbes may contribute to atherosclerotic processes, including *Helicobacter pylori*, *Chlamydia pneumoniae*, cytomegalovirus, hepatitis C virus, and human immunodeficiency virus [37,38]. *M. tuberculosis* may also participate in the development of atherosclerosis. TB infection and atherosclerosis share similar inflammatory processes, which involve increased expression of proinflammatory cytokines (interleukin [IL]-1, IL-2, IL-6, tumor necrosis factor-α, and interferon-γ) and the activation of immune cells (monocytes, macrophage, CD4^+^ T helper 1 [TH1] cells, and TH17 cells) [39]. Previous studies have provided convincing evidence that antibodies against mycobacterial heat-shock protein 65 induce the development of atherosclerosis [40,41]. These findings suggest the involvement of *M. tuberculosis* in atherosclerotic processes that lead to the development of AA and AD.

Ascertainment bias in this study should be considered because patients with TB may be more likely to be diagnosed with AA and AD because of additional chest imaging studies for the workup of TB. To overcome this bias, we conducted stratification analyses of the follow-up periods and the location sites of AA and AD. We found a prolonged risk of AA and AD up to the 5th year after diagnosis. We also found that patients with TB had higher adjusted HRs in both thoracic and abdominal regions than the control participants. These findings confirm that the increased risk of AA and AD in patients with TB is not caused by imaging studies bias.

This study has some limitations. First, NHIRD does not record family history and health-related lifestyle factors in detail, including smoking, body mass index, and alcohol consumption, which were potential confounding factors in this study. Second, relevant clinical variables, including imaging results, acid-fast staining, and culture reports, were unavailable from the database; therefore, we could not confirm the time of TB smear or culture-negativity or assess the severity of TB. Third, the Taiwan Centers for Disease Control did not widely implement latent TB infection screening and treatment until 2016, especially for all age groups with a history of close contact with patients with TB. All cases included in our study between 2000 and 2015 were of active TB infection; therefore, we could not investigate the risk of AA and AD in patients with latent TB. Fourth, bias caused by unknown confounders remains in this retrospective cohort study despite meticulous adjustments. Therefore, a well-designed randomized prospective control study is warranted to conclusively establish a causal relationship between TB and the subsequent development of AA and AD.

## 5. Conclusions

We conducted a nationwide population-based cohort study to investigate the association between TB and the development of AA and AD and analyzed the risk of AA and AD in TB subgroups. Patients with TB had a 1.711-fold higher risk of developing AA and AD than patients without TB, particularly in the miliary TB subgroup. These findings strengthen the association of systemic inflammation between these two diseases. Physicians should consider the subsequent development of AA and AD while treating patients with TB.

## Figures and Tables

**Table 1:** Characteristics of the study participants at baseline.

	Total		With TB		Without TB		*p*
Variables	n	%	n	%	n	%	
Overall	93,660		31,220	33.33	62,440	66.67	
Sex							0.999
Male	66,657	71.17	22,219	71.17	44,438	71.17	
Female	27,003	28.83	9001	28.83	18,002	28.83	
Age (years)	63.67 ± 17.32		63.74 ± 17.33		63.64 ± 17.32		0.405
Age group (years)							0.999
20–44	16,314	17.42	5438	17.42	10,876	17.42	
45–69	33,876	36.17	11,292	36.17	22,584	36.17	
≥70	43,470	46.41	14,490	46.41	28,980	46.41	
Insured premium (NT$)							0.003
<18,000	92,378	98.63	30,768	98.55	61,610	98.67	
18,000–34,999	1032	1.10	385	1.23	647	1.04	
≥35,000	250	0.27	67	0.21	183	0.29	
DM							<0.001
Without	70,368	75.13	23,042	73.81	47,326	75.79	
With	23,292	24.87	8178	26.19	15,114	24.21	
HTN							<0.001
Without	59,003	63.00	21,732	69.61	37,271	59.69	
With	34,657	37.00	9488	30.39	25,169	40.31	
Hyperlipidemia							<0.001
Without	87,769	93.71	30,159	96.60	57,610	92.26	
With	5891	6.29	1061	3.40	4830	7.74	
IHD							<0.001
Without	71,662	76.51	26,219	83.98	45,443	72.78	
With	21,998	23.49	5001	16.02	16,997	27.22	
COPD							<0.001
Without	69,531	74.24	21,719	69.57	47,812	76.57	
With	24,129	25.76	9501	30.43	14,628	23.43	
Stroke							<0.001
Without	73,991	79.00	26,519	84.94	47,472	76.03	
With	19,669	21.00	4701	15.06	14,968	23.97	
CKD							<0.001
Without	89,710	95.78	30,153	96.58	59,557	95.38	
With	3950	4.22	1067	3.42	2883	4.62	
PAOD							0.053
Without	93,603	99.94	31,208	99.96	62,395	99.93	
With	57	0.06	12	0.04	45	0.07	
Obesity							0.002
Without	93,632	99.97	31,218	99.99	62,414	99.96	
With	28	0.03	2	0.01	26	0.04	
Urbanization level							<0.001
1 (The highest)	28,768	30.72	8960	28.70	19,808	31.72	
2	41,696	44.52	13,732	43.98	27,964	44.79	
3	7425	7.93	2565	8.22	4860	7.78	
4 (The lowest)	15,771	16.84	5963	19.10	9808	15.71	

DM = diabetes mellitus, HTN = hypertension, IHD = ischemic heart disease, COPD = chronic obstructive pulmonary disease, CKD = chronic kidney disease, PAOD = peripheral arterial occlusive disease.

**Table 2:** Factors of aortic aneurysm and aortic dissection by using Cox regression.

Variables	Crude HR	95% CI		*p*	aHR	95% CI		*p*
TB								
Without	Reference				Reference			
With	2.064	1.154	2.875	<0.001	1.711	1.098	2.666	<0.001
Sex								
Male	1.501	0.905	2.408	0.110	1.440	0.899	2.306	0.129
Female	Reference				Reference			
Age group (years)								
20–44	Reference				Reference			
45–69	1.786	0.772	2.345	0.512	1.518	0.565	2.103	0.533
≥70	2.065	0.989	3.397	0.668	1.669	0.789	3.020	0.696
Insured premium (NT$)								
<18,000	Reference				Reference			
18,000–34,999	0.000	-	-	0.997	0.000	-	-	0.998
≥35,000	0.000	-	-	0.997	0.000	-	-	0.998
DM								
Without	Reference				Reference			
With	1.589	1.062	2.267	0.020	1.336	0.828	2.154	0.236
HTN								
Without	Reference				Reference			
With	2.309	1.498	3.020	<0.001	1.471	1.311	1.711	<0.001
Hyperlipidemia								
Without	Reference				Reference			
With	0.000	-	-	0.972	0.000	-	-	0.786
IHD								
Without	Reference				Reference			
With	1.903	1.564	2.303	<0.001	1.825	1.555	2.225	<0.001
COPD								
Without	Reference				Reference			
With	0.986	0.387	1.567	0.084	1.087	0.460	1.626	0.067
Stroke								
Without	Reference				Reference			
With	2.976	1.876	4.021	<0.001	2.027	1.690	2.529	<0.001
CKD								
Without	Reference				Reference			
With	1.768	1.001	2.897	0.050	1.563	0.881	2.670	0.323
PAOD								
Without	Reference				Reference			
With	0.000	-	-	0.913	0.000	-	-	0.958
Obesity								
Without	Reference				Reference			
With	0.000	-	-	0.984	0.000	-	-	0.982
Urbanization level								
1 (The highest)	1.726	1.329	2.676	<0.001	1.637	1.278	2.461	<0.001
2	1.682	1.345	2.443	<0.001	1.606	1.259	2.368	<0.001
3	1.201	1.006	1.703	0.045	1.186	0.936	1.694	0.287
4 (The lowest)	Reference				Reference			

HR = hazard ratio, CI = confidence interval, aHR = adjusted hazard ratio: adjusted variables listed in the table, TB = tuberculosis, DM = diabetes mellitus, HTN = hypertension, IHD = ischemic heart disease, COPD = chronic obstructive pulmonary disease, CKD = chronic kidney disease, PAOD = peripheral arterial occlusive disease.

**Table 3:** Incidence and aHR for aortic aneurysm and aortic dissection in the TB and non-TB cohorts stratified by sex, age, socioeconomic status, and comorbidities.

TB	With		Without		With vs. without *(Reference)*							
Variables	Event	Rate (per 10^3^ PYs)	Event	Rate (per 10^3^ PYs)	IRR	95% CI		*p*	aHR	95% CI		*p*
Overall	55	0.185	94	0.150	1.235	1.071	1.567	<0.001	1.711	1.098	2.666	<0.001
Sex												
Male	43	0.202	72	0.161	1.254	1.139	1.631	<0.001	1.738	1.115	2.702	<0.001
Female	12	0.143	22	0.123	1.165	1.032	1.868	0.006	1.616	1.034	2.518	0.008
Age group (years)												
20–44	2	0.061	3	0.051	1.188	1.046	2.976	0.002	1.645	1.051	2.553	0.002
45–69	12	0.125	18	0.098	1.270	1.088	2.000	<0.001	1.760	1.129	2.712	<0.001
≥70	41	0.244	73	0.189	1.287	1.148	1.669	<0.001	1.784	1.153	2.789	<0.001
Insured premium (NT$)												
<18,000	55	0.188	94	0.152	1.238	1.178	1.571	<0.001	1.711	1.098	2.666	<0.001
18,000–34,999	0	0.000	0	0.000	-	-	-	-	-	-	-	-
≥35,000	0	0.000	0	0.000	-	-	-	-	-	-	-	-
DM												
Without	40	0.204	73	0.169	1.208	1.070	1.594	0.003	1.672	1.074	2.609	0.001
With	15	0.149	21	0.108	1.374	1.218	2.036	<0.001	1.911	1.223	2.972	<0.001
HTN												
Without	22	0.141	39	0.139	1.018	1.006	1.541	0.036	1.411	0.995	2.194	0.053
With	33	0.234	55	0.159	1.468	1.347	1.899	<0.001	2.034	1.314	3.287	<0.001
Hyperlipidemia												
Without	55	0.200	94	0.172	1.163	1.057	1.747	<0.001	1.711	1.098	2.666	<0.001
With	0	0.000	0	0.000	-	-	-	-	-	-	-	-
IHD												
Without	35	0.164	52	0.131	1.257	1.105	1.685	<0.001	1.672	1.092	2.585	<0.001
With	20	0.237	42	0.183	1.296	1.198	1.828	<0.001	1.797	1.153	2.801	<0.001
COPD												
Without	31	0.192	68	0.154	1.249	1.098	1.674	<0.001	1.633	1.065	2.598	0.003
With	24	0.177	26	0.141	1.256	1.125	1.810	<0.001	1.740	1.117	2.712	<0.001
Stroke												
Without	37	0.162	57	0.130	1.242	1.069	1.655	<0.001	1.666	1.004	2.635	0.045
With	18	0.264	37	0.196	1.348	1.199	1.911	<0.001	1.869	1.118	3.012	<0.001
CKD												
Without	50	0.178	88	0.148	1.202	1.104	1.549	<0.001	1.675	1.068	2.596	0.007
With	5	0.302	6	0.179	1.681	1.297	2.866	<0.001	2.330	1.486	3.631	<0.001
PAOD												
Without	55	0.185	94	0.150	1.234	1.211	1.581	<0.001	1.711	1.098	2.666	<0.001
With	0	0.000	0	0.000	-	-	-	-	-	-	-	-
Obesity												
Without	55	0.185	94	0.150	1.234	1.194	1.566	<0.001	1.711	1.098	2.666	<0.001
With	0	0.000	0	0.000	-	-	-	-	-	-	-	-
Urbanization level												
1 (The highest)	13	0.163	20	0.113	1.441	1.115	2.139	<0.001	1.997	1.297	3.121	<0.001
2	14	0.110	25	0.087	1.263	1.096	1.917	0.001	1.750	1.125	2.784	<0.001
3	5	0.220	10	0.210	1.048	0.964	2.121	0.067	1.452	0.917	2.266	0.154
4 (The lowest)	23	0.341	39	0.337	1.011	0.646	1.526	0.294	1.400	0.896	2.101	0.297

PYs = person-years, aHR = adjusted hazard ratio: adjusted for the variables listed in Table 2, CI = confidence interval, IRR = incidence rate ratio, TB = tuberculosis, DM = diabetes mellitus, HTN = hypertension, IHD = ischemic heart disease, COPD = chronic obstructive pulmonary disease, CKD = chronic kidney disease, PAOD = peripheral arterial occlusive disease.

**Table 4:** Incidence and aHR for aortic aneurysm and aortic dissection in the TB and non-TB cohorts stratified by TB type.

TB	With		Without		With vs. without *(Reference)*							
TB Subgroup	Event	Rate (per 10^3^ PYs)	Event	Rate (per 10^3^ PYs)	IRR	95% CI		*p*	aHR	95% CI		*p*
Pulmonary TB	42	0.169	94	0.150	1.126	1.058	1.490	0.001	1.561	1.005	2.431	0.044
Extrapulmonary TB	9	0.205	94	0.150	1.365	1.160	2.049	<0.001	1.892	1.214	2.936	<0.001
Miliary TB	4	0.902	94	0.150	6.013	4.870	8.802	<0.001	8.334	5.348	12.896	<0.001

PYs = person-years, aHR = adjusted hazard ratio: adjusted for the variables listed in Table 2; CI = confidence interval, IRR = incidence rate ratio, TB = tuberculosis.

**Table 5:** Incidence and aHR for aortic aneurysm and aortic dissection stratified by different sites.

TB	With		Without		With vs. without *(Reference)*							
AA/AD Subgroup	Event	Rate (per 10^3^ PYs)	Event	Rate (per 10^3^ PYs)	IRR	95% CI		*p*	aHR	95% CI		*p*
Overall	55	0.185	94	0.150	1.235	1.071	1.567	<0.001	1.711	1.098	2.666	<0.001
Thoracic	16	0.054	29	0.046	1.164	1.039	1.498	0.015	1.615	1.044	2.511	0.007
Abdominal	13	0.044	24	0.038	1.143	1.015	1.435	0.038	1.588	1.025	2.469	0.024
Thoracoabdominal	1	0.003	1	0.002	2.110	1.753	2.525	<0.001	2.910	1.876	4.557	<0.001
Unspecified site	25	0.084	40	0.064	1.319	1.188	1.682	<0.001	1.823	1.175	2.843	<0.001

PYs = person-years, aHR = adjusted hazard ratio: adjusted for the variables listed in Table 2, CI = confidence interval, IRR = incidence rate ratio, TB = tuberculosis, AA = aortic aneurysm, AD = aortic dissection.

**Table 6:** Incidence and aHR for aortic aneurysm and aortic dissection stratified by follow-up period.

TB	With		Without		With vs. without *(Reference)*							
Follow-Up Period	Event	Rate (per 10^3^ PYs)	Event	Rate (per 10^3^ PYs)	IRR	95% CI		*p*	aHR	95% CI		*p*
<6 months	17	1.192	7	0.236	5.061	4.267	7.106	<0.001	6.896	5.010	8.226	<0.001
6 months–12 months	4	0.283	5	0.169	1.671	1.335	1.986	<0.001	2.671	1.675	3.145	<0.001
1–5 years	20	0.136	28	0.092	1.481	1.198	1.875	<0.001	2.371	1.486	2.884	<0.001
>5 years	14	0.115	54	0.206	0.560	0.042	0.989	0.041	1.276	0.375	1.790	0.385

PYs = person-years, aHR = adjusted hazard ratio: adjusted for the variables listed in Table 2, CI = confidence interval, IRR = incidence rate ratio, TB = tuberculosis.

## Data Availability

Restrictions apply to the availability of these data. Data was obtained from National Health Insurance database and are available from the authors with the permission of National Health Insurance Administration of Taiwan.
